# Promoting de-implementation of inappropriate antimicrobial use in cardiac device procedures by expanding audit and feedback: protocol for hybrid III type effectiveness/implementation quasi-experimental study

**DOI:** 10.1186/s13012-022-01186-8

**Published:** 2022-01-29

**Authors:** Westyn Branch-Elliman, Rebecca Lamkin, Marlena Shin, Hillary J. Mull, Isabella Epshtein, Samuel Golenbock, Marin L. Schweizer, Kathryn Colborn, Jessica Rove, Judith M. Strymish, Dimitri Drekonja, Maria C. Rodriguez-Barradas, Teena Huan Xu, A. Rani Elwy

**Affiliations:** 1 Center for Healthcare Organization and Implementation Research, VA Boston Healthcare System, Boston, USA; 2 Department of Medicine, Infectious Disease Section, VA Boston Healthcare System, Boston, USA; 3 Harvard Medical School, Boston, USA; 4 Department of Surgery, Boston University School of Medicine, Boston, USA; 5 Iowa City VA Health Care System, Iowa, USA; 6 University of Iowa, Iowa, USA; 7 Eastern Colorado Healthcare System, Aurora, USA; 8 Department of Surgery, University of Colorado Anschutz Medical Campus, Denver, USA; 9 Infectious Diseases Section, Minneapolis VA Healthcare System, Minneapolis, USA; 10 Michael E. DeBakey VAMC, Houston, Texas USA; 11 Baylor College of Medicine, Houston, Texas USA; 12 Center for Healthcare Organization and Implementation Research, VA Bedford Healthcare System, Bedford, USA; 13 Department of Psychiatry and Human Behavior, Warren Alpert Medical School, Brown University, Providence, USA

**Keywords:** De-implementation, Learning/unlearning, i-PAHRIS, Stepped wedge, Hybrid type III implementation/effectiveness, Antimicrobial stewardship, Informatics

## Abstract

**Background:**

Despite a strong evidence base and clinical guidelines specifically recommending against prolonged post-procedural antimicrobial use, studies indicate that the practice is common following cardiac device procedures. Formative evaluations conducted by the study team suggest that inappropriate antimicrobial use may be driven by information silos that drive provider belief that antimicrobials are not harmful, in part due to lack of complete feedback about all types of clinical outcomes. De-implementation is recognized as an important area of research that can lead to reductions in unnecessary, wasteful, or harmful practices, such as excess antimicrobial use following cardiac device procedures; however, investigations into strategies that lead to successful de-implementation are limited. The overarching hypothesis to be tested in this trial is that a bundle of implementation strategies that includes audit and feedback about direct patient harms caused by inappropriate prescribing can lead to successful de-implementation of guideline-discordant care.

**Methods:**

We propose a hybrid type III effectiveness-implementation stepped-wedge intervention trial at three high-volume, high-complexity VA medical centers. The main study intervention (an informatics-based, real-time audit-and-feedback tool) was developed based on learning/unlearning theory and formative evaluations and guided by the integrated-Promoting Action on Research Implementation in Health Services (i-PARIHS) Framework. Elements of the bundled and multifaceted implementation strategy to promote appropriate prescribing will include audit-and-feedback reports that include information about antibiotic harms, stakeholder engagement, patient and provider education, identification of local champions, and blended facilitation. The primary study outcome is adoption of evidence-based practice (de-implementation of inappropriate antimicrobial use). Clinical outcomes (cardiac device infections, acute kidney injuries and *Clostridioides difficile* infections) are secondary. Qualitative interviews will assess relevant implementation outcomes (acceptability, adoption, fidelity, feasibility).

**Discussion:**

De-implementation theory suggests that factors that may have a particularly strong influence on de-implementation include strength of the underlying evidence, the complexity of the intervention, and patient and provider anxiety and fear about changing an established practice. This study will assess whether a multifaceted intervention mapped to identified de-implementation barriers leads to measurable improvements in provision of guideline-concordant antimicrobial use. Findings will improve understanding about factors that impact successful or unsuccessful de-implementation of harmful or wasteful healthcare practices.

**Trial registration:**

ClinicalTrials.govNCT05020418

Contributions to the literature

- De-implementation is an important, but understudied area. Factors that promote de-implementation require learning and unlearning opportunities and are different than those that promote implementation.
- This theory-driven, multicenter effectiveness-implementation trial assesses whether a bundled implementation strategy that specifically targets increasing providers’ knowledge and awareness of risks and benefits of antimicrobial prophylaxis through a novel informatics-based audit-and-feedback tool can promote practice change.
- This study will provide insight into whether strategies that target information gaps caused by differential feedback to providers about clinical outcomes that directly result from their interventions, specifically, guideline-discordant antimicrobial use, can promote de-implementation of common, yet harmful, practices.

## Introduction

In the USA, more than 300,000 cardiovascular implantable electronic devices (CIEDs), such as pacemakers and defibrillators, are placed annually [1, 2]. Guideline-driven antimicrobial prophylaxis reduces CIED infections and improves outcomes. Clinical guidelines emphasize the importance of timing and duration of peri-procedural prophylaxis to achieve optimal benefit [3, 4]. To reduce infections, antimicrobials must be given before incision. Discontinuation within 24 h after skin closure is recommended because prolonged antimicrobials cause harm, including acute kidney injuries (AKI) and *Clostridioides difficile* infections, but do not reduce infections [3, 5, 6, 7].

Guidelines about timing and duration of antimicrobial prophylaxis are often not adhered to, and excessive antimicrobials are prescribed after more than 50% of CIED procedures, leading to patient harms [8]. Formative evaluations with electrophysiologists who perform these procedures concluded that these clinicians are highly motivated to prevent infections but that they overestimate benefits of prolonged antimicrobial use and underestimate harms, in part due to lack of feedback about the adverse consequences that result from prolonged antimicrobial prophylaxis [9].

De-implementation is recognized as an important area of research that can lead to reductions in unnecessary, wasteful, or harmful clinical practices, such as excess antimicrobial use following CIED procedures; however, investigations into approaches that lead to successful de-implementation are limited [10]. Theories suggest that factors that may have a particularly strong influence on de-implementation include strength of the underlying evidence, the complexity of the intervention, and patient and provider anxiety and fear about changing an established practice [11]. Because de-implementation is a distinct process [12], tailored strategies that recognize de-implementation specific barriers may be necessary to achieve successful discontinuation [11].

To promote de-implementation of excess antimicrobial use following CIED procedures in Veterans Health Administration (VA) medical centers, our team devised a hybrid type III effectiveness-implementation trial with a quasi-experimental design (NCT05020418) involving a multi-faceted implementation strategy that was developed based on theories of learning/unlearning and de-implementation to close the information gap. The multi-faceted implementation intervention was guided by formative evaluations and includes (1) an innovative clinical informatics-based semi-automated surveillance system that will be used to audit and provide feedback to providers about guideline-concordance and cardiac and non-cardiac outcomes (CIED infections [13], AKI, *C. difficile*); (2) a multi-level education intervention that targets prescribers, other clinical providers, and patients; (3) engagement of local champions; and (4) blended facilitation and local adaptation. The overarching hypothesis of this study is that information gaps and fear about adverse consequence contribute to a lack of acceptance of guidelines and continuation of guideline-discordant practices.

We outline our protocol for this de-implementation trial, specifying the conceptual model driving current practice, the theory guiding our work, our de-implementation framework, de-implementation strategies, and specific measures to assess the effectiveness of our strategies as well as our clinical outcomes. A key hypothesis that will be tested is if provider motivation to optimize clinical outcomes can be leveraged using a multi-faceted implementation bundle that incorporates audit-and-feedback about benefits and harms of current practice to decrease guideline-discordant care.

### Conceptual model: information silos and gaps in feedback

**Fig. 1:**
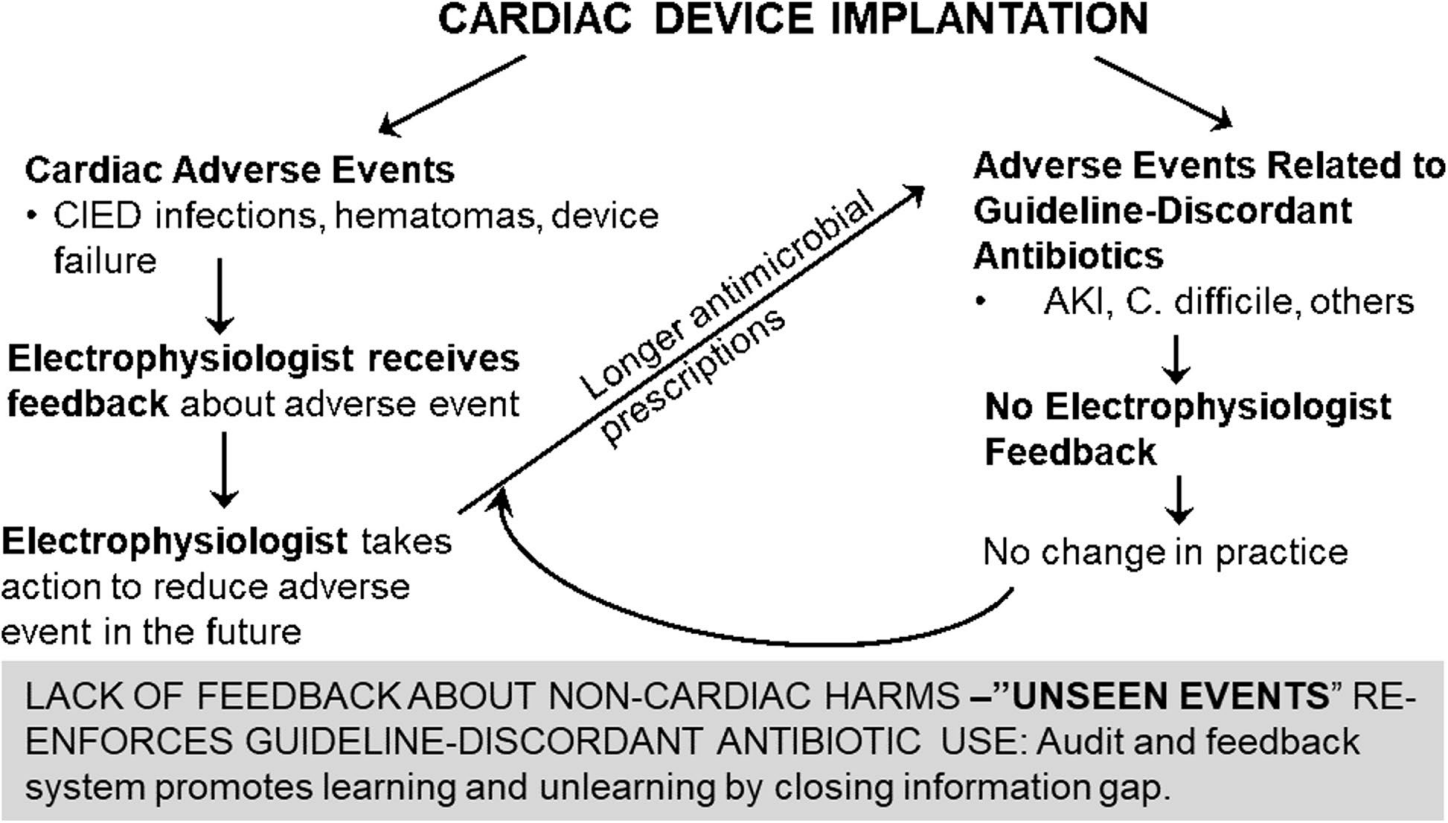
Conceptual model of information feedback loops, how they reinforce delivery of guideline-discordant care, and how they influence clinical decision-making

Clinicians often do not see the “whole clinical picture” of a patient because they receive incomplete data (Fig. 1, Conceptual Model). Electrophysiologists are often acutely aware of certain adverse outcomes, such as severe CIED infections, but unaware of non-cardiac adverse outcomes that result from their practices that none-the-less cause patient harm, such as *C. difficile* infections and AKI. This lack of communication between providers (siloing of information) leads to gaps in feedback that impact and re-enforce physician behavior [14, 15]. This conceptual model was supported by formative evaluations, which found that electrophysiologists are highly motivated to prevent complications related to their specific area of clinical practice, such as cardiac device infections, but are less motivated to avoid other types of adverse events, in part because they do not learn about them and in part because they do not feel responsible for them, and therefore these harms are not considered in their clinical decision making. Additional barriers that must be overcome in order to achieve de-adoption in this clinical context include (1) lack of knowledge about the adverse consequences of antimicrobial over use and confidence stemming from their clinical experiences; (2) a desire to streamline processes across a facility; (3) a desire to provide a perceived “standard of care,” which incorrectly includes guideline-discordant care; and (4) concern that stopping antimicrobials at the time of skin closure might increase CIED infections and that these infections would not be detected, due to a lack of surveillance systems for outpatient and procedural settings [9].

### Theoretical basis: learning and unlearning

De-implementation is a two-phased process that requires unlearning one practice and learning another [16]. According to current understanding of learning/unlearning, hereafter referred to as unlearning, persuasive strategies, including education, use of local champions, and audit and feedback are effective for promoting complete discontinuation of an ineffective practice, as is necessary for successful de-implementation [16–18]. Persuasive strategies are known to improve de-adoption of inappropriate antimicrobial use in inpatient units [19]. In the context of unlearning, Whittington et al. [20] propose three types of audit and feedback strategies: *informational feedback* (to fill *knowledge gaps*), *evaluative feedback* (to *motivate* providers who are aware the practice is low-value but are not adopting the change), and *suggestive feedback* (to replace an ineffective practice with a more effective one).

### Implementation framework

The multi-faceted implementation strategy was developed using the integrated-Promoting Action on Research Implementation in Health Services (iPARIHS) framework [21], which emphasizes the importance of Innovation, Recipients, Context, and Facilitation. Within *Innovation*, the evidence quality and its relationship to current practices are important considerations. Motivation, values, goals, knowledge, resources, and support are key factors of *Recipients*. *Context* includes both local and external factors. *Facilitation* assesses and responds to “characteristics of the innovation and the recipients within their contextual setting.” [21] In this project, *external facilitation* will be achieved through centralized data collection and analysis by the study team. *Local facilitation* will be achieved through the activities of champions, who will interact with electrophysiology team members to effect practice change at the participating sites. Each component of the iPARIHS framework is paired to implementation strategies (Table 1).

**Table 1:** Relationship between iPARIHS Constructs and the Research Plan and Selected Multi-Faced Implementation Strategy

iPARIHS construct		Relationship to research plan/implementation strategies
Innovation	Underlying knowledge	Evidence supporting prevention practices is strong, as evidenced by inclusion of recommendations for pre-incisional prophylaxis with early discontinuation in guidelines endorsed by multiple societies and guideline-issuing bodies. The strength of the evidence supports the viability of a program designed to facilitate uptake of proven effective antimicrobial use.
	Compatibility	There are limited local resources dedicated to surveillance and other prevention activities. Our study design, using a centralized automated system with adjudication and validation at a central site, bypasses these resource restraints.
	Usability	Local practice patterns and variability may impact how surveillance reports are used; thus, a 6-month local adaptations and piloting phase is included to enhance usability and facilitate uptake. Sites will also have the opportunity to provide feedback about usability and operability of the electronic data monitoring tool at their own site, and to request local adaptations.
	Observable results	All variables that will be included in the tool are extractable from the rich VA EHR. Variables that will be extracted electronically include number of procedures with and without guideline-concordant pre-procedure prophylaxis and guideline-discordant post-procedure prophylaxis and facility rank, 90-day CIED infection rate and facility rank, 7-day incidence of acute kidney injury, 90-day incidence of *C. difficile* infection. Manual review will be used to augment the electronic data pull, and qualitative analysis will be used to enrich the quantitative data.
Recipients	Motivation, values, and beliefs	Clinicians express a desire to ensure their patients have the best outcomes. This implementation strategy highlights the safety of stopping antimicrobials- and that stopping antimicrobials improves the overall health of their patients by (1) not increasing risk of infection, and (2) decreasing the incidence of patient-level antibiotic-associated adverse events (e.g., acute kidney injuries, *C. difficile* infections, others).
	Time, resources, support	Data will be collected through a centralized, automated detection and reporting system with manual adjudication performed at the primary study site. Because local resources and IT support are not required, time, resources, and support required to use the centralized system are low for the participating sites and champions.
	Local opinion leaders, Policy Makers	Infectious diseases champions are drivers of practice change and local protocol changes. To leverage the importance of these knowledge leaders, we have the support of policy-making organizations. In addition, process and outcome reports will be provided to local champions to facilitate practice improvements.
Context	Culture	Electrophysiologists commonly conform to local culture about prevention strategies and express concerns about being an “outlier.” Benchmarking is included to demonstrate that the provider is not “an outlier.”
	Evaluation and feedback	Audit and feedback reports will be used to demonstrate to providers that a transition to guideline-concordant recommendations is not harmful to patients, and in fact, improves care.
Facilitation	Internal	Local champions will provide input into fidelity-consistent modifications to the audit and feedback reports. Local content experts will serve as facilitators of change, using the data provided and leveraging their status as content and thought leaders. These local experts are also able to write and change local protocols and thus mandate a larger local culture change.
	External	External facilitation will involve education, central data collection and adjudication of output from the electronic surveillance algorithm, adjustments to the algorithm based on feedback from sites to improve accuracy and data analysis. The aims of the external facilitation will be to reduce the local burden of surveillance, feedback, and development of educational materials on the intervention sites such that implementation is feasible.

### Implementation strategies

The multi-faceted implementation strategy to be tested in this hybrid type III quasi-experimental trial will include an informative component and an evaluative component to integrate an unlearning process with known barriers to de-implementation. The *informative component* will include feedback and educational sessions about prevention of CIED infections, the effectiveness of pre- and post-procedural antimicrobials, and information about antimicrobial harms. This will be provided through educational sessions to electrophysiology teams, blended facilitation, and direct feedback from local champions. The *evaluative component* will include reports provided to facilities with information about adverse events and rates of guideline-concordant care with benchmarking.

## Study design and methods

### Trial design

This clinical trial uses a hybrid type III effectiveness-implementation stepped-wedge design at three high-volume and high-complexity VA medical centers (Table 2) [22]. The primary endpoint evaluated in hybrid type III studies relates to the effectiveness of the implementation strategy or strategies; clinical outcomes are secondary [22]. Increased uptake of guideline-based recommendations is expected to translate into improved clinical outcomes; however, the study is primarily powered to assess the effectiveness of the multi-faceted intervention for promoting adoption of guideline-concordant antimicrobial use practices.

**Table 2:** Stepped-wedge design of the hybrid III implementation/effectiveness trial

Site	0–6 Months	6–12	12–15	15–18	18–21	21–24	24–30	30–46	36–48
**1**	Development of educational materials, IRB submission, data collection, initial report development	Education	Adaptation, facilitation	Wash-in	Reports provided	Reports provided	Reports provided	Reports provided	Qualitative data and quantitative collection and analysis
**2**			Education	Adaptation, facilitation	Wash-in	Reports provided	Reports provided	Reports provided	
**3**				Education	Adaptation, facilitation	Wash-in	Reports provided	Reports provided	

The stepped-wedge design is chosen for several reasons. Stepped-wedge designs have been used successfully in implementation research trials [23, 24], including trials designed to improve infection prevention and patient safety practices [25]. The stepped-wedge design staggers the introduction of the implementation strategy. Because of this, stepped-wedge trials are susceptible to changes based on secular trends; however, by temporally aligning sites awaiting implementation with sites undergoing active implementation, stepped-wedge trials have the advantage of more fully controlling for practice trends unrelated to the implementation strategy than alternative designs, such as parallel-groups randomized controlled trials [26].

### Elements of the multi-faceted implementation strategy (main study interventions)

The multi-faceted implementation strategy is informed by processes of unlearning [15, 17], the iPARIHS framework, and leverages strategies that address known barriers to de-implementation. Previous research demonstrates that multi-faceted implementation strategy bundles lead to the delivery of more evidence-based treatment and that use of multiple implementation strategies is a predictor of programmatic success [27]. Centralized, semi-automated surveillance will address barriers identified during formative evaluations. The benefits of guideline-concordant practice will be communicated through education and reinforced through other iPARIHS constructs, including local adaptations and blended facilitation. Internal facilitation will be performed by local champions, and external facilitation by study team will occur through centralized data collection and dissemination. As indicated in Table 3, the strategy is informed by previously identified barriers and facilitators of de-adoption specific to the cardiac electrophysiology laboratory. The six major elements of the multi-faceted implementation strategy follow.

**Table 3:**
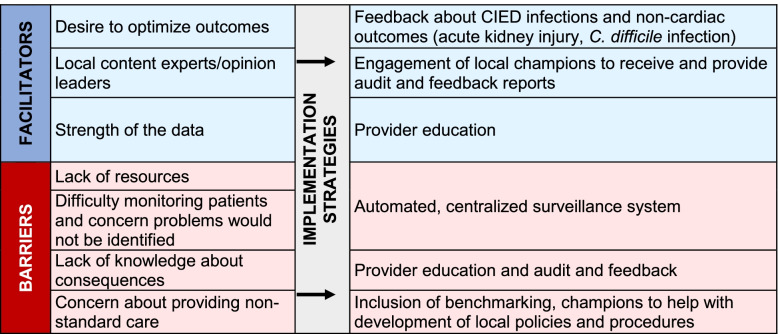
Barriers and facilitators to de-adoption identified during formative evaluations and how they inform the multi-faceted implementation strategy for hybrid III implementation/effectiveness stepped-wedge study

#### Quality monitoring/audit and feedback reports

Reports created by the primary site will be provided to local champions (i.e., infectious diseases/infection control team members) via a secured data transfer method. Reports will include data such as (1) rates of appropriate pre-procedural antimicrobial use, (2) CIED infection events and rates, (3) antimicrobial-associated adverse events and rates (e.g., *C. difficile* infections, AKIs), (4) rates of post-procedural antimicrobial use benchmarked to high-performing facilities and providers, and (5) CIED infection rates benchmarked to other facilities. Rates of guideline-concordant antimicrobial use are included to reinforce positive behavior (learning), and rates of guideline-discordant use and benchmarking are included to promote unlearning and de-adoption. The reports will give the sites information about application of guideline-concordant practices at other high-performing facilities to ensure providers that they are not “outliers,” a barrier identified in formative evaluations. Rates of infections and adverse events are included to provide reassurance that the practice change is improving clinical outcomes by reducing non-cardiac adverse events and not worsening CIED infection rates. Adverse events that arise from inappropriate antimicrobial use are included to highlight tangible harms associated with lack of guideline compliance and to promote *unlearning*.

#### Local adaptations to reports to optimize performance and local utility

Facilities will be provided with preliminary audit and feedback reports, which will include CIED infection rates calculated using the electronic data extraction tool followed by manual validation, rates of guideline-concordant pre-procedural antimicrobial use and guideline-discordant post-procedural antimicrobial use, AKIs and *C. difficile* infections, and benchmarking about post-procedural antimicrobial use to high-performing sites. Local feedback will be used to make adaptations, such as the preferred method and timing for receiving the report, the preferred method of data presentation and comparison (graphs, quantitative measures), and the degree to which individualized versus facility-level data are included. Sites will be asked to provide input about the accuracy of the reports (e.g., were there infection cases identified by clinical operations that were not detected?), the utility of the reports (e.g., were the cases known to the infectious diseases and infection control services) and about how to make the reports user-friendly. Input from the participating sites will be incorporated and reports will be revised based on their feedback. If necessary, based on feedback about accuracy of infection detection, adjustments to the electronic algorithm will be made to improve performance. Investigators will track all feedback, including about accuracy and changes to the electronic detection tool, in the final report of findings.

#### Identification and engagement of key stakeholder groups

Several key stakeholder groups have been identified and will be engaged during the project. These include cardiology stakeholders (e.g., electrophysiologists and other electrophysiology team members) and infectious diseases and infection control stakeholders (e.g., infectious diseases physicians and pharmacists, infection control specialists). *Electrophysiologists* and other cardiology stakeholders are targeted by the educational and feedback components of the strategy. *Infectious diseases/infection control specialists* are the local champions and internal facilitators who will act upon the reports.

#### Identification of local champions and clinical experts

Champions at the three intervention facilities will participate as clinical leads at the three intervention sites. Formative evaluations identified that local champions, such as local infectious diseases specialists and antimicrobial stewardship and infection control experts, are major drivers of sustained practice improvement, consistent with published data [28, 29]. These champions are the ideal *internal facilitators* for promoting practice improvement for several reasons. First, reducing infections and improving antimicrobial use are central aspects of their clinical and administrative duties [7, 30, 31]. These champions have protected clinical and administrative time dedicated to improving antimicrobial use [32] and are passionate about promoting the safe and effective use of antimicrobials [33]. Second, as part of their clinical and administrative rules, local antimicrobial stewardship content experts are empowered to revise local protocols and CIED infection prevention guidelines. However, they do not have the necessary technological support to measure rates of CIED infection or compliance with guideline-concordant practice and thus have limited resources to promote change; the automated audit and feedback system included as part of the implementation intervention addresses this critical need.

#### Education

Education of both providers and patients will be included. Educational sessions for providers will provide information about (a) clinical guidelines, (b) harms associated with guideline non-compliance, and (c) the audit and feedback reports. Educational sessions will be scheduled to coincide with standing conferences, such as grand rounds, infection prevention committee, and/or morbidity and mortality, to enhance attendance and knowledge about the program and its goals. Patient education materials will be developed and distributed; these will be developed by a graphic medicine specialist and will include information about the risks and benefits of peri-procedural antimicrobial use and things that a patient can do to prevent and identify infections.

#### Blended facilitation

Facilitation is a critical aspect of the iPARIHS framework and will be addressed internally and externally (i.e., blended). The preliminary results highlight the degree to which current practice is determined by normative factors; thus, providing feedback about the overall shift in clinical practice and resulting outcomes may promote de-adoption of guideline-discordant practice by limiting provider concerns about “being an outlier” and medical malpractice concerns associated with not adhering to the locally entrenched, but not evidence-based, standard of care. The implementation strategy external facilitation will be achieved through in-person or virtual educational sessions, centralized data collection and verification through reports, and site check-ins (either virtually or in-person, depending upon what is feasible given the pandemic) that will encourage participation and allow progress to be reviewed. Internal facilitation will be achieved by local champions, who will provide educational sessions, present the data to providers, encourage uptake of evidence-based practices, and collaborate with electrophysiology team members to develop local policies and procedures.

##### Study setting

Three large volume, VA medical centers were selected based on (1) high volume of procedures (e.g., >100 procedures per year), (2) high rates of inappropriate antimicrobial use (>50%), and (3) operating characteristics of all elements of the semi-automated surveillance tools during the development and validation of the electronic monitoring tool that will be used for the audit-and-feedback reports [13, 34].

##### Study design

A stepped-wedge implementation/effectiveness trial will be conducted at three VA medical centers.

##### Study timeline

An overview of the stepped-wedge process is presented in Table 2. The implementation intervention will be rolled out at different sites at different times and will occur in several phases.

#### Phases of the stepped/wedge design

##### Phase I: educational sessions and feedback from sites about audit and feedback reports

Educational seminars and/or webinars developed by the coordinating site and provided to clinical infectious diseases champions at the intervention sites will be presented to electrophysiology team members. Educational sessions will focus on benefits of pre-procedural antimicrobial prophylaxis, harms of post-procedural antimicrobial prophylaxis, and strategies for reducing infection. The semi-automated audit and feedback surveillance system with site-specific data available on a secure dashboard will also be introduced.

##### Phase II: wash-in, initiation of locally adapted reports

Prior to providing the audit and feedback reports, the local champions will be asked to provide input about the accuracy of the semi-automated algorithm for detecting true adverse events and about usability and feasibility. Based on feedback, the surveillance tools and reports will be locally adapted to each of the participating study sites. After local adaptation, sites will be provided with monthly surveillance reports, which will be manually validated by the primary study site. Champions at each site will be contacted and informed that the reports are available and ready for review via a protected and shared server with capabilities to monitor frequency of report access (measure of site fidelity to the intervention).

##### Phase III: reports provided

Monthly reports developed on previously published semi-automated electronic data extraction algorithms [34] that include concordance with guideline-based antimicrobial use practices at the facility and benchmarked to practices from the entire VA healthcare system, cardiac device infections and rate, and non-cardiac adverse events (AKI, *C. difficile* infections) will be provided to the local champions via the secured shared site. Thus, the information will be available for monthly conferences. Sites that do not access the reports for a 2-month period will be contacted by email to encourage report access and use (i.e., external facilitation). If email is not effective, sites will be contacted via phone and local infectious diseases champions will be asked about the lapse, support will be offered, and use encouraged. Intermittent site visits (either virtual given the pandemic or in-person) will also be used to encourage participation.

##### Phase IV: data analysis

During the last year of the project, qualitative and quantitative summative data will be analyzed. Quantitative data will be analyzed to measure *adoption* (through change in proportion of cases with guideline-concordant practice) and *fidelity* (measurement of report access). Quantitative data about clinical endpoints (*C. difficile* infections, AKI) will be extracted from electronic health records (EHRs) and analyzed. The wash-in period will be excluded. Qualitative will be collected through semi-structured interviews (in-person or virtually) with key stakeholders.

#### Trial outcomes and assessments of effectiveness

This study will collect quantitative and qualitative data. The primary outcomes are implementation outcomes, with clinical outcomes secondary.

### Implementation outcomes

#### Primary outcome (implementation outcome, adoption of evidence-based practice)

The primary outcome measure of the hybrid type III trial is the change in the proportion of cases with guideline-concordant antimicrobial discontinuation within 24 h after skin closure, which is the guideline-recommended and evidence-based practice. Antimicrobial use patterns pre- and post-implementation will be measured using the previously described automated algorithm for measuring peri-operative antimicrobial prophylaxis, which has >**97%** accuracy at the three participating sites. The adoption and maintenance of guideline-concordant pre-procedure prophylaxis and de-adoption of guideline-discordant post-procedure prophylaxis will be measured quantitatively as a change in proportion of procedures with guideline-concordant practice using longitudinal data from three VA study sites. Assessments of audit and feedback report access and use by the study sites will be performed to ensure that the reports are being accessed and used as a measure of fidelity. Specifically, implementation fidelity will be measured quantitatively using access to reports and will be measured by calculating the number of months reports were available/number of months reports were accessed; sites will then be assigned a fidelity rank score (high, moderate, low) based on report access.

Additional information about the effectiveness of the multi-faceted implementation strategy for other implementation outcomes (e.g., feasibility, fidelity, acceptability) will be collected during qualitative interviews of key stakeholders at each of the three sites.

### Clinical outcomes

#### Non-cardiac adverse events

##### Laboratory-defined AKI and *C. difficile*

A *C. difficile* infection is defined as a positive stool test from the initial date of antimicrobial exposure to within 90 days following the last day of antimicrobial prophylaxis. AKI is defined as occurring from the initial date of antimicrobial exposure to within 7 days of the last dose of antimicrobial prophylaxis and is based on AKIN-Network definitions. AKI severity will be reported (stages I, II, or III).

#### Cardiac adverse events

Ninety-day incidence of cardiac device infections will be measured using the semi-automated measurement tool adapted for near-real time surveillance. Effectiveness of the tool based on champion feedback will be included in trial results.

### Data analysis

#### Analysis of quantitative data elements

The primary outcome measure is the *adoption* of guideline-based pre-procedure prophylaxis and *de-adoption* of guideline-discordant post-procedure prophylaxis, defined as a change in the proportion of procedures with guideline-concordant antimicrobial use practices. This will be assessed by using an interrupted time-series analysis at the three study sites with comparison of proportions, accounting for facility and random fixed-effects. A regression model adjusted for patient and facility characteristics will then be used to estimate the impact of fidelity of accessing reports on adoption of guideline-based practices.

The quantitative effectiveness (primary outcome) of the implementation strategy will be measured through the change in the adoption of evidence-based antimicrobial use at the three sites. This will be calculated as the change in proportion of cases *without* post-procedural antimicrobials lasting for >24 h after skin closure, as measured by the automated algorithm. The analysis will use a generalized linear mixed model to estimate the probability of pre-and post-implementation antimicrobial use, accounting for facility-level correlation as a random effect. We will also explore the influence of calendar time in the Stepped-Wedge Design using models described by Nickless et al. [35].

#### Clinical outcomes are secondary in this hybrid type III trial

Clinical outcomes reported are selected to assess the impact of improving adoption of guideline-concordant antimicrobial prophylaxis on important clinical outcomes theoretically linked to appropriate application of peri-operative prophylaxis, specifically, CIED infections (impacted by appropriate pre-procedural antimicrobial use), and AKI and *C. difficile infections* (both impacted by appropriate early discontinuation of antimicrobial use). These data will be extracted from the VA EHR using the automated algorithm and CIED infections will be validated by manual review at the main study site. Similar to the analysis of the implementation outcomes, incidence of key clinical outcomes will be measured pre- and post-implementation at each of the three sites, applying a generalized linear mixed model with allowance for a facility random effect. We will again explore the influence of calendar time in the stepped-wedge design using models described by Nickless et al. [35].

### Power calculations

#### Adoption of evidence-based practices (primary outcome)

Using the power estimation procedure of Hussey and Hughes [36], we will need to evaluate 135 cases from each site, inclusive of before and after the surveillance system is active, and setting alpha = 0.05, we will have 80% power to detect a difference in proportion greater than or equal to 0.15. In other words, if the rate of guideline discordant antimicrobial use in an individual facility changed from 50 out of 100 procedures to 35 out of 100 at each site after introduction of our audit and feedback system, we could detect a significant change in proportion. We assumed a coefficient of variation equal to 0.2 for this estimate. The three facilities perform >100 CIED procedures per year and all have rates of guideline discordant antimicrobial use following > 50% of procedures.

#### Impact on CIED infections outcomes (secondary)

Based on estimated incidence of CIED infections and *C. difficile*, and the number of procedures performed across the three facilities (*N*~2100), the study has 89% power to detect a doubling in the incidence of CIED infections (from 2 to 4%) and 50% power to detect a 50% reduction in infections (from 2 to 1%). These estimates were again derived from the formulas of Hussey and Hughes and assumed a coefficient of variation of 0.2 [36].

#### Qualitative Data Collection and Analysis

Qualitative data will be collected through semi-structured interviews with key stakeholders using interview guides developed using the iPARIHS framework. Interview guides will contain questions related to feasibility, acceptability, and future adaptations to ensure that important implementation outcomes are represented.

Summative evaluations at each of the study sites will elicit electrophysiology team members’ perspectives about how reports impacted clinical practice decisions. Interviews with electrophysiologists will explore whether the information supplied in the reports was acceptable and useful and whether reports lead to practice change. Electrophysiologists will also be asked which elements of the multi-faceted implementation strategy impacted their practice the most, how the audit and feedback contributed to change (e.g., unlearning), and about fidelity to guidelines. Data from interviews with infectious diseases and infection control team members will focus on feasibility, and adaptations and will be analyzed separately. Infection control and antimicrobial stewardship specialists will be asked about any changes to local processes, protocols, or procedures that may have resulted from the implementation strategy and for input regarding which elements of the strategy they found to be the most useful (acceptability), and for any suggestions for how to improve it for future adaptation and dissemination. If changes to local policies were made, investigators will request protocol documents from before and after the change and they will be compared and classified according to the type of change (e.g., new EHR order set, new facility policy, etc.). If a facility demonstrates limited or no positive change, semi-structured interviews will explore why electrophysiology teams found the strategy to be ineffective and potential future adaptations will be identified. If there is differential effectiveness across sites, facilities with high levels of de-implementation will be compared to facilities with low levels of de-implementation to identify potential factors that may have impacted success, including acceptability, feasibility, and cost factors. Information about how the reports impacted clinical care, including information about unintended consequences, will be collected.

#### Qualitative data analysis plan

Video or audio-recordings will be transcribed and coded using qualitative analytic software. Transcripts will be initially coded using a priori constructs consistent with our conceptual model, relevant implementation outcomes (i.e., acceptability, adoption, fidelity, feasibility), and iPARIHS, which will be outlined in a codebook with definitions and examples. A directed content analysis approach with allowance for new themes to emerge will be used [37].

The qualitative data analysis will be conducted by at least three study investigators and inter-rater reliability will be established using the “check-coding” process [38]. All coders will independently code the same interview transcripts. Coders will then meet to compare their coding, discuss areas of difficulty, and reach agreement on the definitions and examples in the codebook. A new interview will then be independently coded by all, and the process will be repeated until coders achieve a mutual understanding of the domain definitions and when to apply the codes [38]. Upon completion of coding, we will summarize data in matrix displays utilizing Miles and Huberman’s analytical approaches to help compare and contrast data across sites [39]. Thereafter, site-specific descriptive summaries, which will include key information that can be used to summarize findings, will be produced.

#### Discussion and limitations of the approach

This study will focus on de-implementing an intervention with a strong evidence basis against its use, as well as a guideline-based recommendation supporting the practice change. This is both a strength and a limitation of the study. Prior research and the formative interviews suggest that the strength of the knowledge base is a major driver of success [11]. This factor is likely to increase the probability of this project’s success, however, will limit generalizability to settings where the evidence base and the clinical guidelines are less clear, particularly to interventions with a mixed or untested evidence base. In addition, this study is targeting a relatively simple clinical intervention—short-term prescribing of a single medication; simple and short-term interventions are generally viewed as easier to de-implement than more complex interventions. The use of external facilitation and a semi-automated surveillance system reduce the workload burden on intervention sites; limiting personnel requirements at the participating sites is another factor that favors success of this specific project but may limit generalizability to interventions that would require facilities to hire additional staff to carry out the proposed interventions. In addition, this study will be conducted at three large volume VA medical centers. Findings may not be generalizable to other VA medical centers or to non-VA medical systems. Finally, this study uses a quasi-experimental, rather than randomized, design. This will impact our ability to fully attribute causality to the multifaceted implementation bundle.

### Summary

The multi-faceted implementation strategy that will be tested in the hybrid type III study will include audit and feedback using a novel computer-based algorithm, education, engagement of local champions, blended facilitation, and local adaptation. If effective, similar approaches to promoting de-implementation could be adapted for other clinical and non-clinical settings where lack of communication across disciplines, limited feedback to providers about a range of clinical outcomes, and concerns about adverse impacts of de-adoption drive inappropriate, wasteful, or harmful practices.

## Data Availability

Not applicable.

## Abbreviations

VA: Veterans affairs
iPARHIS: Integrated-Promoting Action on Research Implementation in Health Services Framework
CIED: Cardiovascular Implantable Electronic Device
AKI: Acute kidney injury
EHR: Electronic Health Record
